# Analysis of the correlation between the degree of sleepiness and the therapeutic effect in patients with insomnia disorder treated with digital cognitive behavioral therapy

**DOI:** 10.3389/fpsyt.2026.1849522

**Published:** 2026-06-22

**Authors:** Yangfei Che, Mingfen Song, Lili Yang, Junhang Zhang, Hongjing Mao

**Affiliations:** 1 The Fourth Clinical Medical College of Zhejiang Chinese Medical University, Hangzhou, China; 2 Affiliated Mental Health Centre & Hangzhou Seventh People’s Hospital, Zhejiang University School of Medicine, Hangzhou, China

**Keywords:** daytime sleepiness, digital cognitive behavioral therapy, Epworth sleepiness scale, insomnia disorder, Pittsburgh Sleep Quality Index (PSQI)

## Abstract

**Introduction:**

Some patients experience increased daytime sleepiness during early dCBT‑I, which may affect function and adherence. This study aimed to investigate daytime sleepiness during dCBT‑I, explore its influencing factors, and evaluate its predictive value for treatment efficacy.

**Methods:**

A retrospective analysis was conducted on 2271 insomnia patients treated at the sleep clinic of Hangzhou Seventh People’s Hospital from November 2016 to January 2024. Daytime sleepiness and sleep quality were assessed using the Epworth Sleepiness Scale (ESS) and Pittsburgh Sleep Quality Index (PSQI).

**Results:**

ESS scores significantly increased at week 2, 4, and 6, peaking at week 2. Sex, medication use, snoring, and baseline ESS were significant influencing factors. Patients with early increased sleepiness showed greater PSQI reduction rates at subsequent follow‑ups. Univariate and multivariable linear regression confirmed that week 2 ESS exacerbation independently predicted higher PSQI reduction at weeks 4, 8, 12, and 16 (all p < 0.05).

**Discussion:**

Transient increased daytime sleepiness often occurs in the early stage of dCBT‑I, which is related to multiple factors and can serve as a predictor of subsequent treatment efficacy.

## Introduction

Insomnia is one of the most common sleep disorders (1, 2). Insomnia disorder refers to the frequent and persistent difficulty in falling asleep or maintaining sleep despite having sufficient sleep opportunities and a suitable environment, resulting in insufficient sleep satisfaction and affecting daytime function or causing physical discomfort. These symptoms occur at least three times per week and persist for 3 months or longer (3). Long-term insomnia may lead to a series of physical and mental health problems, such as fatigue, daytime sleepiness, emotional instability, and difficulty in concentrating (4), seriously affecting patients’ daily work, quality of life, and functions such as immunity and metabolism (5, 6).

Traditional treatment methods for insomnia include drug treatment and behavioral therapy. Drug treatment can quickly relieve the clinical symptoms of insomnia, but it is associated with potential adverse reactions, and long-term use may increase the risk of tolerance and dependence in patients (7, 8). In recent years, cognitive behavioral therapy for insomnia (CBT-I) has been proven to be an effective nonpharmacological intervention for treating insomnia (9, 10). The Chinese guidelines (9) recommend cognitive behavioral therapy for insomnia disorder as an important first-line treatment. The main treatment components of CBT-I include sleep education, sleep restriction therapy, stimulus control therapy, relaxation training, and cognitive therapy (11). It combines cognitive therapy and behavioral therapy and can relieve the difficulty in falling asleep, improve sleep quality, and maintain long-term efficacy (12).

Although CBT-I has a significant effect on improving sleep quality, traditional CBT-I relies on one-on-one contact and conversation between professional therapists and patients to provide personalized treatment and has several disadvantages, such as complex processes, high economic costs, and a relatively limited number of professional therapists. Therefore, with the development of Internet technology, digital cognitive behavioral therapy for insomnia (dCBT-I) has been proposed and has gradually attracted attention. dCBT-I mainly provides various forms of treatment content through online platforms, such as a series of structured video courses that explain sleep hygiene knowledge, sleep restriction principles, and relaxation training in detail (13). Some studies have also confirmed that dCBT-I is effective in treating insomnia disorder (14–16).

However, clinical observations and some studies (17) have revealed that some patients may experience an increased daytime sleepiness in the early stage of CBT-I treatment, which may have a negative impact on the patients’ daytime function and treatment adherence. Reports on daytime sleepiness in patients during dCBT-I treatment are relatively rare. This study aimed to clarify whether patients experience increased daytime sleepiness during dCBT-I treatment, identify the time of sleepiness onset, analyze influencing factors, and explore its impact on overall efficacy. In addition, this study analyzes whether increased daytime sleepiness can serve as a potential predictor of dCBT-I efficacy, thereby providing a basis for optimizing the treatment strategies.

## Materials and methods

### Participants

Patients diagnosed with insomnia disorder who visited the Sleep Clinic of Hangzhou Seventh People’s Hospital between November 2016 and January 2024 were selected.

### Inclusion and exclusion criteria

The inclusion criteria were as follows: (1) meeting the diagnostic criteria for insomnia disorder in the Diagnostic and Statistical Manual of Mental Disorders, fifth edition (DSM-5) (18); (2) having a score greater than 7 on the Pittsburgh Sleep Quality Index (PSQI); (3) having at least baseline assessment data and assessment data from any one of the remaining observation points; and (4) patients and their family members being informed about this study.

The exclusion criteria were as follows: (1) suffering from other sleep disorders, such as restless legs syndrome, obstructive sleep apnea hypopnea syndrome, narcolepsy, etc.; (2) suffering from severe physical diseases; (3) suffering from severe mental diseases (such as bipolar disorder and obsessive-compulsive disorder); (4) having any significant disorders of perception, memory, or behavior; (5) lacking baseline data; (6) having fewer than two assessments at each observation point (including baseline data); (7) among patients with snoring, those with a clear diagnosis of obstructive sleep apnea hypopnea syndrome were excluded, and only those with simple snoring and occasional snoring were included. After screening, a total of 2,271 patients met the inclusion criteria and did not meet the exclusion criteria.

### Detection measures

#### Assessment of daytime sleepiness by the Epworth Sleepiness Scale

The Epworth Sleepiness Scale (ESS) (19) evaluates daytime sleepiness by using scores of sleepiness levels in eight situations to reflect an individual’s degree of daytime sleepiness. The score range is from 0 to 3 points, representing “impossible to doze off” to “very likely to doze off”, respectively. The total score ranged from 0 to 24 points, and the higher the total score, the greater the degree of daytime sleepiness.

#### Assessment of sleep status by the Pittsburgh Sleep Quality Index

The PSQI (20) is a widely used self-assessment sleep quality scale, mainly used to assess sleep quality and the severity of sleep disorders. The scale contains seven dimensions, including sleep quality, sleep latency, sleep duration, sleep efficiency, sleep disturbances, use of sleep medications, and daytime dysfunction. The score range of each dimension is 0–3 points, and a total score ranging from 0 to 21 is generated cumulatively. The higher the total score, the worse the sleep quality. Generally, a PSQI score > 5 points is regarded as an indicator of sleep disorders.

#### Digital cognitive behavioral therapy for insomnia

dCBT-I was completed by patients through the “Good Sleep 365” smartphone platform. Good Sleep 365 is a mobile application designed for patients with insomnia and has been described in our previous publications (21–23). The content included the following: (1) Stage assessment: patients conducted an initial assessment on the app at the first visit to obtain baseline data, and assessments were conducted every 2 weeks in the early stage of treatment, that is, at weeks 2, 4, 6, and 8, and then every 4 weeks thereafter, that is, at weeks 12 and 16. The assessment scales included the ESS scale and the PSQI scale. (2) Relaxation training: the app customizes and pushes “relaxation training” based on the patient’s scale assessment results and the situation at the time of visit to help patients relax and fall asleep without drugs. (3) Sleep restriction therapy: the app determines bedtime in line with sleep restriction principles according to the patient’s daily bedtime habits, or it can be jointly negotiated by the doctor and the patient in the clinic according to the patient’s situation, with bedtime generally not exceeding 7 h. (4) Push of other CBT-I modules: the app pushes content such as “sleep hygiene, stimulus control, and cognitive restructuring” in different sequences according to the assessment results and the most prominent sleep problems of the patient.

### Participants

SPSS 23.0 software was used for data analysis to compare the total ESS score at each observation point with the baseline score. A *t*-test was used to compare the PSQI score reduction rates between the two groups of patients with and without a peak in ESS score after treatment. Spearman correlation analysis was used to examine the correlation between the occurrence of a peak in the ESS score and various influencing factors. To investigate the relationship between week 2 ESS score exacerbation and treatment outcomes, univariate and multivariable linear regression analyses were performed with PSQI reduction rates at weeks 4, 8, 12, and 16 as dependent variables, respectively, and “ESS score exacerbation at week 2” as the independent variable. Univariate linear regression was first conducted without adjustment for other covariates. Subsequently, sex, age, disease duration, education level, and baseline PSQI were entered using the Enter method as covariates to construct four separate multivariable linear regression models to adjust for potential confounders. For all regression analyses, unstandardized coefficients (*B*), 95% confidence intervals (CI), and *p*-values are reported.

### Ethical approval

Due to the retrospective nature of the study, the ethics committee of the Institutional Review Board of Hangzhou Seventh People’s Hospital (approval number: 2024-044) waived the need to obtain informed consent. The experimental protocol was approved by the ethics committee of the Institutional Review Board of Hangzhou Seventh People’s Hospital, and all methods and procedures were performed in accordance with relevant guidelines and regulations.

## Results

### Patient data

Baseline data of 2,271 cases were recorded. After excluding cases with no assessment at the current observation point and cases in which the total number of assessments (including one baseline data) was less than two, according to the inclusion and exclusion criteria, the retained patient data samples were 1,341 cases at week 2, 1,328 cases at week 4, 1,191 cases at week 6, 1,485 cases at week 8, 1,393 cases at week 12, and 1,157 cases at week 16.

### Change in daytime sleepiness

**Figure 1:**
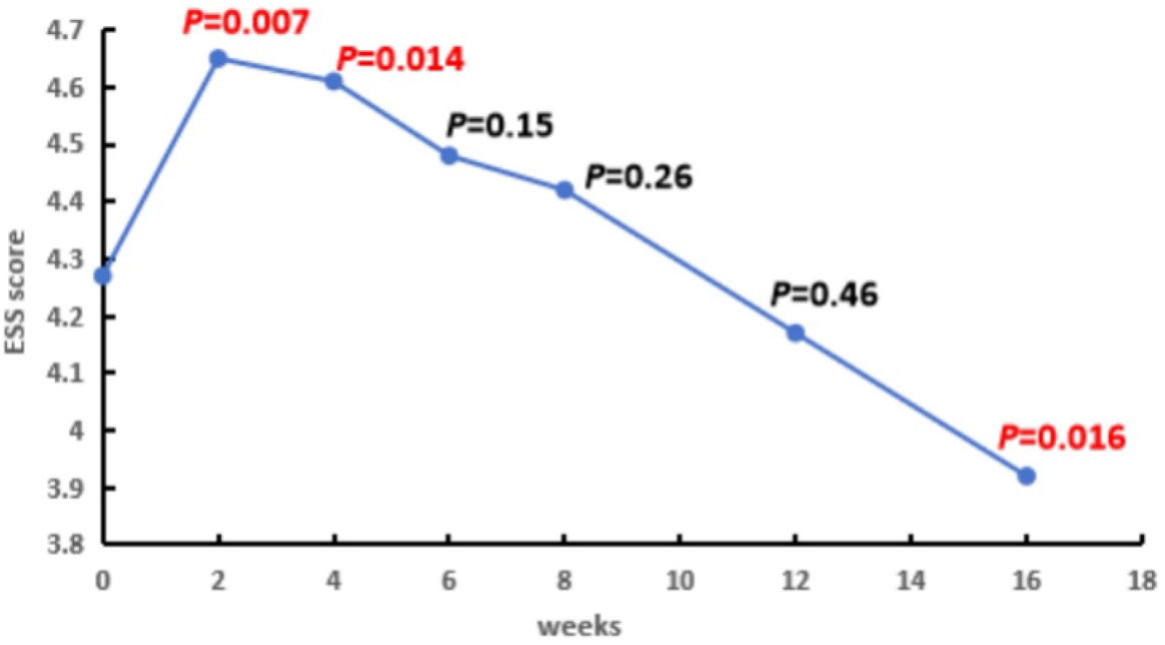
Changes in daytime sleepiness in patients before treatment and during dCBT-I treatment at weeks 2, 4, 6, 8, 12, and 16. Baseline, total ESS score of the patient before treatment; ESS, Epworth Sleepiness Score.

Comparing the total ESS score at weeks 2, 4, 6, 8, 12, and 16 with the baseline score, the overall score was *F* = 4.514. The total ESS score of patients increased at weeks 2, 4, 6, and 8 after receiving dCBT-I treatment, indicating an increase in daytime sleepiness. It returned to the pretreatment level at week 12 and then began to decrease. The increase was most obvious at week 2 (*p* = 0.007), which represented the peak of the total ESS score. The score returned to the pretreatment level at week 12 and decreased significantly at week 16 (*p* = 0.016). The differences between each time point and the baseline are shown in **Figure 1**.

A comparison of demographic factors was conducted between patients who experienced an increase in daytime sleepiness and those who did not at week 2. Since the basic information of eight patients was not available, a total of 1,333 patients were included in the analysis. The *t*-test was used to analyze multiple demographic characteristics of the two groups of patients, including sex, age, body mass index (BMI), education level, disease duration, past medication use, baseline medication use, family history, snoring status, and baseline ESS score. The results (**Table 1**) revealed that sex (*p* = 0.042) and the baseline ESS score (*p* < 0.001) were significantly different between the two groups of patients. Other characteristics, such as age (*p* = 0.937), BMI (*p* = 0.402), education level (*p* = 0.984), disease duration (*p* = 0.163), past medication use (*p* = 0.764), current medication use (*p* = 0.097), and family history (*p* = 0.128), were not significantly different between the two groups. **Table 1** provides details. Spearman analysis was also used to conduct a correlation analysis of the factors influencing whether there was an increase in sleepiness at week 2. According to the results (**Table 2**), factors such as patient sex (*p* = 0.009), baseline medication use (*p* < 0.001), snoring (*p* = 0.007), and the baseline ESS score (*p* < 0.001) were significantly correlated with increased daytime sleepiness at week 2. Patient sex and the baseline ESS score were negatively correlated with increased daytime sleepiness at week 2, whereas baseline medication use and snoring were positively correlated with increased daytime sleepiness at week 2. **Table 2** provides details.

**Table 1:** Comparison of demographic characteristics according to exacerbation drowsiness in the second week.

Characteristics	The degree of drowsiness worsened in the second week	The level of drowsiness remains unchanged in the second week	t	P-value
Sex				
Male	161 (25.8)	218 (30.8)	4.129	0.042
Female	464 (74.2)	490 (69.2)		
Age (years)	45.06 ± 12.18	45.01 ± 13.15	0.08	0.937
BMI (kg/m2)	21.57 ± 2.53	21.40 ± 13.15	0.84	0.402
Educational level				
Primary school	72 (11.5)	79 (11.2)	0.158	0.984
Middle school	294 (47.0)	329 (46.5)		
University	224 (35.9)	258 (36.4)		
Postgraduate	35 (5.6)	42 (5.9)		
Course of disease				
< 3 months	126 (20.2)	176 (24.9)	6.524	0.163
3–12 months	124 (19.8)	128 (18.1)		
1–3 years	114 (18.2)	142 (20.0)		
3–5 years	78 (12.5)	73 (10.3)		
5–10 years	183 (29.3)	189 (26.7)		
Previous medication				
No	191 (30.6)	211 (29.8)	0.090	0.764
Yes	434 (69.4)	497 (70.2)		
Baseline medication				
No	263 (42.1)	330 (46.6)	2.759	0.097
Yes	362 (57.9)	378 (53.4)		
Family history				
No	496 (79.4)	585 (82.6)	2.311	0.128
Yes	129 (20.6)	123 (17.4)		
Snore				
No	373 (59.7)	437 (61.7)	0.581	0.446
Yes	252 (40.3)	271 (38.3)		
Baseline ESS score	3.17 ± 3.31	5.09 ± 4.57	8.85	< 0.001

**Table 2:** Correlation analysis of factors influencing whether drowsiness worsened in the second week.

Demographic characteristics	Correlation coefficient	P-value
Sex (0 female, 1 male)	− 0.136	0.009
Age (year)	0.062	0.235
BMI (kg/m2)	0.068	0.194
Educational level	0.009	0.863
Course of disease	− 0.021	0.684
Previous medication (no/yes)	0.072	0.169
Baseline medication (no/yes)	0.215	< 0.001
Family history (no/yes)	0.050	0.176
Snore (no/yes)	0.141	0.007
Baseline ESS	− 0.368	< 0.001

### Changes in sleep parameters

**Figure 2:**
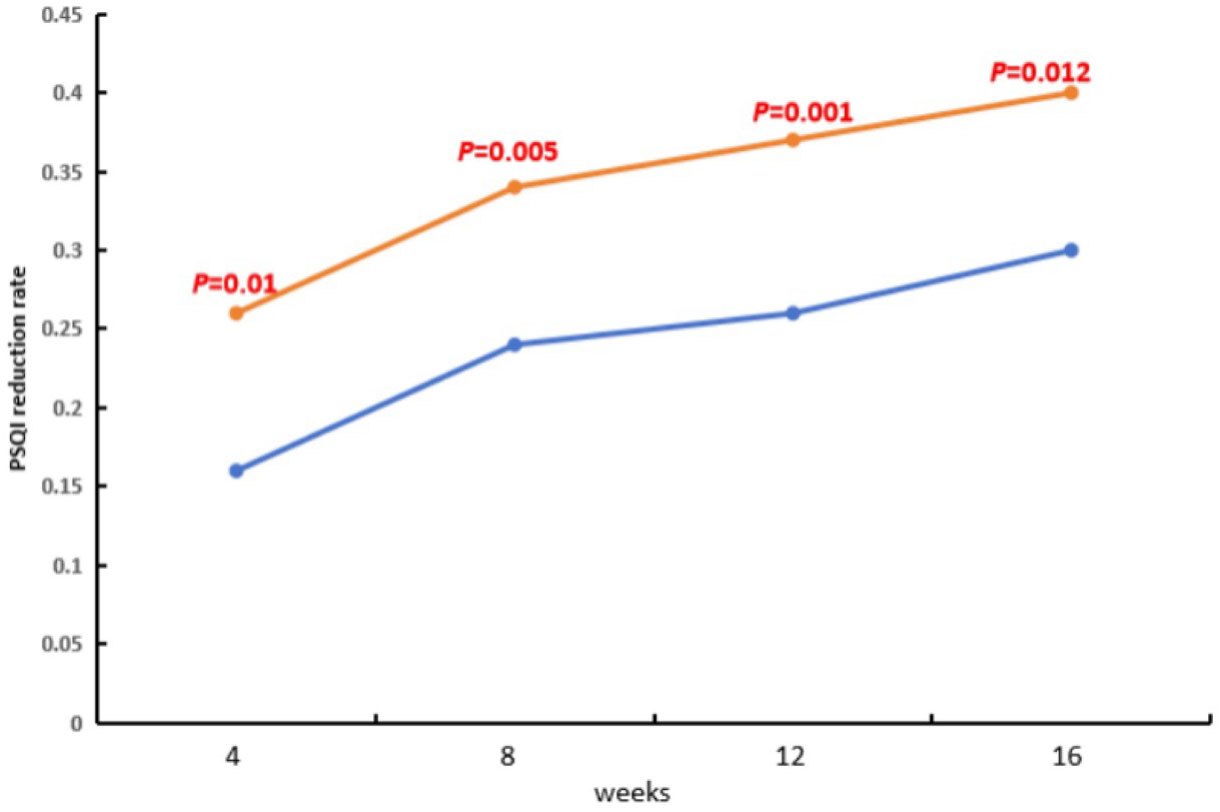
Changes in PSQI score reduction rate of patients at weeks 4, 8, 12, and 16 after receiving networked dCBT-I treatment.

A *t*-test was conducted on the rate of decrease in the PSQI score of patients with a peak total ESS score (week 2) and those without increased daytime sleepiness at week 2. The sleep quality improvement in patients with increased daytime sleepiness at week 2 was greater than that in patients without increased daytime sleepiness at weeks 2 at 4 (*t* = 2.59; *p* = 0.010), week 8 (*t* = 2.86; *p* = 0.005), week 12 (*t* = 3.210; *p* = 0.001), and week 16 (*t* = 2.53; *p* = 0.012) after treatment. The PSQI score reduction rate values at each time point are shown in **Table 3**, and the comparison of the PSQI score reduction rate at each time point is shown in **Figure 2**.

**Table 3:** Comparison of PSQI score reduction at each observation point between patients who experienced increased daytime sleepiness in the second week after receiving dCBT-I and those who did not.

Patient subgroup (ESS score change at week 2)	Fourth week reduction rate	Sixth week reduction rate	Twelfth week reduction rate	Sixteenth week reduction rate
Total ESS score in the second week ≤ 0	0.16 ± 0.41 (0.032)	0.24 ± 0.37 (0.029)	0.26 ± 0.395 (0.031)	0.30 ± 0.39 (0.031)
Total ESS score in the second week > 0	0.26 ± 0.23 (0.018)	0.34 ± 0.21 (0.017)	0.37 ± 0.23 (0.019)	0.40 ± 0.26 (0.021)

To further quantify the association, univariate linear regression analyses were performed with week 2 ESS exacerbation as the independent variable and PSQI reduction rates at weeks 4, 8, 12, and 16 as dependent variables. The results confirmed that week 2 ESS exacerbation was significantly positively associated with PSQI reduction at week 4 (*B* = 0.010; 95% CI: 0.024–0.173; *p* = 0.010), week 8 (*B* = 0.098; 95% CI: 0.031–0.165; *p* = 0.005), week 12 (*B* = 0.118; 95% CI: 0.045–0.190; *p* = 0.002), and week 16 (*B* = 0.096; 95% CI: 0.021–0.170; *p* = 0.012). These univariate results are consistent with the *t*-test findings and provide regression coefficients that indicate the magnitude of the effect. Relevant values at each time point are presented in **Table 4**.

**Table 4:** Univariate linear regression of week 2 ESS exacerbation on PSQI reduction rates.

Time point	B	95% CI	P-value
Week 4	0.098	[0.024, 0.173]	0.010
Week 8	0.098	[0.031, 0.165]	0.005
Week 12	0.118	[0.045, 0.190]	0.002
Week 16	0.096	[0.021, 0.170]	0.012

Furthermore, to evaluate the independent predictive role of week 2 increased sleepiness, we constructed multivariable linear regression models for the PSQI reduction rates at weeks 4, 8, 12, and 16, respectively, adjusting for sex, age, disease duration, education level, and baseline PSQI. The results are shown in **Table 5**. At week 4, week 2 ESS exacerbation was significantly positively associated with PSQI reduction rate (*B* = 0.081; 95% CI: 0.008–0.154; *p* = 0.029); education level (*B* = 0.079; *p* = 0.004) and baseline PSQI (*B* = 0.025; *p* < 0.001) were also significant. At week 8, week 2 ESS exacerbation still significantly predicted a higher PSQI reduction rate (*B* = 0.091; 95% CI: 0.024–0.157; *p* = 0.008), with education level (*B* = 0.059; *p* = 0.018) and baseline PSQI (*B* = 0.020; *p* < 0.001) remaining significant. At week 12, week 2 ESS exacerbation maintained a significant positive association (*B* = 0.105; 95% CI: 0.034–0.157; *p* = 0.004), and education level (*B* = 0.083; *p* = 0.002) and baseline PSQI (*B* = 0.022, *p* < 0.001) were also significant. At week 16, week 2 ESS exacerbation was again significant (*B* = 0.089; 95% CI: 0.016–0.163; *p* = 0.017), and education level (*B* = 0.091; *p* = 0.001) and baseline PSQI (*B* = 0.019; *p* = 0.001) remained independent predictors. In all four models, sex, age, and disease duration were not statistically significant (all *p* > 0.05). These results consistently demonstrate that, after adjustment for demographic factors and baseline insomnia severity, week 2 increased sleepiness remains an independent positive predictor of subsequent treatment efficacy.

**Table 5:** Multivariable linear regression of week 2 ESS exacerbation on PSQI reduction rates at each follow-up time point.

Dependent variable	Independent variable	B	95% CI	Beta	P-value
PSQI reduction rate at week 4	ESS exacerbation	0.081	[0.008, 0.154]	0.120	0.029
	Sex	0.002	[− 0.079, 0.082]	0.002	0.965
	Age	0.001	[− 0.002, 0.004]	0.033	0.579
	Course of disease	0.020	[− 0.004, 0.045]	0.092	0.106
	Educational level	0.079	[0.025, 0.133]	0.168	0.004
	Baseline PSQI score	0.025	[0.013, 0.036]	0.232	< 0.001
PSQI reduction rate at week 8	ESS exacerbation	0.091	[0.024, 0.157]	0.147	0.008
	Sex	0.054	[− 0.019, 0.127]	0.081	0.148
	Age	− 7.2210−5	[− 0.003, 0.003]	− 0.003	0.963
	Course of disease	0.021	[− 0.001, 0.044]	0.106	0.064
	Educational level	0.059	[0.010, 0.109]	0.139	0.018
	Baseline PSQI score	0.020	[0.009, 0.030]	0.203	< 0.001
PSQI reduction rate at week 12	ESS exacerbation	0.105	[0.034, 0.177]	0.159	0.004
	Sex	0.018	[− 0.061, 0.096]	0.025	0.657
	Age	0.002	[− 0.001, 0.005]	0.065	0.280
	Course of disease	0.010	[− 0.014, 0.034]	0.046	0.418
	Educational level	0.083	[0.030, 0.136]	0.181	0.002
	Baseline PSQI score	0.022	[0.011, 0.033]	0.211	< 0.001
PSQI reduction rate at week 16	ESS exacerbation	0.089	[0.016, 0.163]	0.132	0.017
	Sex	0.023	[− 0.058, 0.104]	0.031	0.575
	Age	0.001	[− 0.002, 0.004]	0.034	0.578
	Course of disease	0.001	[− 0.024, 0.026]	0.005	0.927
	Educational level	0.091	[0.036, 0.145]	0.193	0.001
	Baseline PSQI score	0.019	[0.008, 0.031]	0.181	0.001

## Research discussion

This study conducted a systematic analysis of dynamic changes in daytime sleepiness among patients with insomnia receiving dCBT-I, based on large-sample real-world data. It clarified the temporal pattern, key influencing factors, and correlation with therapeutic efficacy, thereby providing an important basis for clinical interpretation of early treatment responses.

First, this study confirmed that increased daytime sleepiness represents a common transient reaction in the early stage of dCBT-I, which is consistent with the findings of some previous studies (24). It occurs mainly between 2 and 6 weeks, with the peak at week 2, gradually subsiding thereafter, returning to baseline at week 12, and being significantly lower than baseline at week 16. This pattern indicates that daytime sleepiness is not a treatment failure or adverse effect but an adaptive manifestation during the remodeling of sleep architecture and circadian rhythm. This phenomenon is likely related to sleep restriction therapy (SRT) (25, 26), a core component of dCBT-I. SRT (27, 28) improves sleep efficiency by reducing time in bed, which increases homeostatic sleep drive but may lead to relative short-term sleep deprivation. Meanwhile, SRT requires fixed bed and wake-up times to reset the circadian clock, which is a gradual process and may cause temporary circadian misalignment and disturbed sleep–wake rhythms, collectively leading to daytime sleepiness. As treatment proceeds, sleep efficiency improves, and circadian rhythm stabilizes; consequently, sleepiness is naturally relieved (29).

Second, we compared patients with and without increased daytime sleepiness at week 2. The results showed that sex, baseline medication use, snoring, and baseline ESS score were significantly associated with increased daytime sleepiness at week 2. Female sex (30, 31), baseline medication use, snoring, and a lower baseline ESS score were associated with a higher risk of early increased daytime sleepiness. Baseline medication use may contribute to residual sedative effects (32), and snoring may indicate increased upper airway resistance or occult respiratory events (33, 34), which manifest more prominently as daytime sleepiness during sleep restructuring. These factors can be used for early clinical risk identification to improve patient education and treatment adherence.

More importantly, beyond the *t*-test comparisons, univariate linear regression analyses confirmed that week 2 ESS exacerbation was significantly positively associated with PSQI reduction rates at weeks 4, 8, 12, and 16 (all *p* < 0.05). Furthermore, multivariable linear regression models adjusting for sex, age, disease duration, education level, and baseline PSQI consistently demonstrated that week 2 ESS exacerbation remained an independent positive predictor of PSQI reduction at all four time points (all *p* < 0.05). These findings provide stronger evidence that early increased sleepiness reflects a clear physiological response to sleep restriction and circadian alignment, representing an external sign of activated therapeutic mechanisms rather than an adverse signal (35). The persistence of the association after adjustment for multiple confounders supports the robustness of this predictive value. Patients who experience sleepiness in the initial stage should not discontinue treatment, as this phenomenon predicts better subsequent sleep improvement (36).

From a clinical perspective, our results support the safety and effectiveness of dCBT-I (13). Early daytime sleepiness is mostly a temporary and adaptive reaction that can be alleviated through adequate patient education, regular schedules, and fixed wake-up times. Stratified management based on sex, medication use, snoring, and baseline ESS helps optimize intervention, reduce dropout rates, and improve overall outcomes.

In conclusion, increased daytime sleepiness in the early stage of dCBT-I is a predictable, explainable, and tolerable benign response associated with better therapeutic effects. Early increased sleepiness serves as an independent predictor of subsequent treatment efficacy. Clinical practice should strengthen patient education and follow-up, use early changes in sleepiness as a reference for efficacy monitoring, and promote the standardized and wider application of digital therapy in insomnia management.

## Conclusion

This study shows that dCBT-I is an effective and safe treatment method for patients with insomnia disorder. The degree of daytime sleepiness in patients increased during the second, fourth, and sixth weeks of treatment. Among these, the second week was the peak period when most patients experienced an increase in daytime sleepiness. Moreover, the degree of daytime sleepiness gradually returned to the pretreatment level starting from the 12th week of treatment and significantly decreased by the 16th week.

At the same time, we found that whether there is an increase in daytime sleepiness during the second week is closely related to several factors, such as gender, the patient’s current medication use, snoring, and the baseline ESS score. Specifically, female patients, those with a lower baseline ESS score, those who are not currently taking medications for the treatment of insomnia disorder, and those who do not snore during sleep are more likely to experience an increase in daytime sleepiness in the second week.

For the group of patients with increased daytime sleepiness, the improvement rates of their sleep conditions at the fourth, eighth, 12th, and 16th weeks were significantly faster compared with the group of patients without increased daytime sleepiness during the second week. Furthermore, univariate linear regression analyses confirmed that week 2 ESS exacerbation was significantly positively associated with PSQI reduction at weeks 4, 8, 12, and 16 (all *p* < 0.05). Multivariable linear regression models adjusting for sex, age, disease duration, education level, and baseline PSQI further demonstrated that week 2 ESS exacerbation remained an independent positive predictor of PSQI reduction at all four time points (all *p* < 0.05). The phenomenon of increased daytime sleepiness during the second week of dCBT-I treatment can serve as an independent predictive reference index for the efficacy of dCBT-I.

## Limitations

First, this was a single-center retrospective real-world study, and all participants were enrolled from the sleep clinic of Hangzhou Seventh People’s Hospital. Therefore, selection bias may exist, and the generalizability of the results to other regions and populations needs to be further validated.

Second, daytime sleepiness and sleep quality were evaluated using subjective self-rated scales (ESS and PSQI) without objective sleep monitoring data, such as polysomnography (PSG) or actigraphy, which might lead to measurement bias.

Third, patients completed dCBT-I independently via a mobile application. Individual differences in treatment adherence, completion rates, and learning progress were not quantitatively recorded or controlled, which may have impacted the observed outcomes.

## Data Availability

Due to confidentiality agreements with participants, the data are not publicly available. Data supporting the findings of this study are available from the corresponding author upon reasonable request.
